# Aflatoxin Contamination of Non-cultivated Fruits in Zambia

**DOI:** 10.3389/fmicb.2019.01840

**Published:** 2019-08-09

**Authors:** Paul W. Kachapulula, Ranajit Bandyopadhyay, Peter J. Cotty

**Affiliations:** ^1^USDA-ARS Aflatoxin Laboratory, School of Plant Sciences, The University of Arizona, Tucson, AZ, United States; ^2^Plant Pathology Laboratory, School of Agricultural Sciences, Department of Plant Science, University of Zambia, Lusaka, Zambia; ^3^International Institute of Tropical Agriculture (IITA), Ibadan, Nigeria

**Keywords:** aflatoxin, *Aspergillus*, *Schinziophyton*, non-cultivated fruits, Zambia, food safety

## Abstract

Wild fruits are an important food and income source for many households in Zambia. Non-cultivated plants may be as susceptible as crops to aflatoxin contamination. Concentrations of aflatoxins in commonly consumed wild fruits from markets and characteristics of associated aflatoxin-producers need to be determined to assess the aflatoxin risk posed by handling, processing, storage, and consumption. Samples of *Schinziophyton rautanenii* (*n* = 22), *Vangueriopsis lanciflora* (*n* = 7), *Thespesia garckeana* (*n* = 17), *Parinari curatellifolia* (*n* = 17), *Ziziphus* spp. (*n* = 10), *Adansonia digitata* (*n* = 9), and *Tamarindus indica* (*n* = 23) were assayed for aflatoxin using lateral-flow immunochromatography from 2016 to 2017. Aflatoxins were above Zambia’s regulatory limit (10 μg/kg) in *S. rautanenii* (average = 57 μg/kg), *V. lanciflora* (average = 12 μg/kg), and *T. garckeana* (average = 11 μg/kg). The L strain morphotype of *Aspergillus flavus* was the most frequent member of *Aspergillus* section *Flavi* in market samples, although *Aspergillus parasiticus* and fungi with S morphology were also found. All fruits except *T. indica* supported both growth (mean = 3.1 × 10^8^ CFU/g) and aflatoxin production (mean = 35,375 μg/kg) by aflatoxigenic *Aspergillus* section *Flavi*. Innate resistance to aflatoxin producers was displayed by *T. indica*. For the other fruits, environment and infecting fungi appeared to have the greatest potential to influence aflatoxin concentrations in markets. This is the first report of aflatoxins and aflatoxin-producers on native fruits in Zambia and suggests mitigation is required.

## Introduction

Wild fruits supplement diets and incomes of people in portions of rural Zambia (Kalaba et al., 2009). These fruits are gathered for direct consumption, especially during famines (Akinnifesi et al., 2008), and for sale in urban centers (Kalaba et al., 2009). More than 75 wild fruit species are consumed in Southern Africa (Akinnifesi et al., 2002; Kalaba et al., 2009), with some of the popular fruits being *Parinari curatellifolia*, *Ziziphus mauritiana*, *Schinziophyton rautanenii, Uapaca kirkiana*, and *Anisophyllea boehmii* (Zimba et al., 2005; Chadare et al., 2008; Kalaba et al., 2009). Wild fruits have various uses (Table 1) including processing into juice and porridge. Fruit seeds may be eaten as a snack or extracted for oil (Juliani et al., 2007; Chadare et al., 2008; Maroyi, 2011; Vermaak et al., 2011; Njana et al., 2013; Benhura et al., 2015; Rahul et al., 2015; Maruza et al., 2017). Consumption of wild fruits is expected to increase and efforts to domesticate wild fruit tree species are increasing (Akinnifesi et al., 2002; Akinnifesi et al., 2006). Approval of fruits as food ingredients by the European Commission and the U.S. Food & Drug Administration has increased demand for wild fruits, e.g., *Adansonia digitata*, in the western world and is expected to outstrip supply (Buchmann et al., 2010; Buchwald-Werner and Bischoff, 2011). Food safety concerns associated with wild fruits affect consumers in both rural and urban areas.

**TABLE 1:** Uses of non-cultivated fruits frequently sold in local markets in Zambia.

**Fruit species**	**Common name**	**Uses**	**References**
*Adansonia digitata*	Baobab	Fruit pulp used to make beverages, porridges. Leaves, bark, and seeds medicinal	Chadare et al., 2008; Rahul et al., 2015; Vermaak et al., 2011
*Parinari curatellifolia*	Hissing tree, mobola plum, cork tree	Fruit pulp used to make beverages, porridges. Leaf extracts medicinal. Seeds eaten as snacks and used to extract oil	Akinnifesi et al., 2006; Benhura et al., 2015
*Schinziophyton rautanenii*	Mongongo, manketti	Fruit pulp used to make beverages, porridges. Seeds eaten as snacks and used to extract oil	Zimba et al., 2005; Vermaak et al., 2011
*Tamarindus indica*	Tamarind	Fruit pulp used to make beverages, porridges. Leaves are used in medicines and used in feed	Ebifa-Othieno et al., 2017
*Thespesia garckeana*	Snot apple	Fruit pulp used to make beverages, porridges. Medicinal	Maroyi, 2011
*Vangueriopsis lanciflora*	False wild medlar, crooked false medlar	Fruit pulp used to make beverages, porridges.	
*Ziziphus* spp.	Black date, Chinese date, date seed, and several others	Beverages, jams, cakes, medicinal	Maruza et al., 2017

Wild fruits will likely remain a vital component of diets and, as such, it is important to ensure these fruits are free of hazardous microbes and mycotoxins, such as aflatoxins (Boyd and Cotty, 2001). Consumption of food contaminated with aflatoxins can lead to liver cancer, immuno-suppression, stunting, reduced weight-gain, and rapid death in humans (Turner et al., 2003; Gong et al., 2004; Williams et al., 2004; Lewis et al., 2005; Probst et al., 2007; Reddy and Raghavender, 2007; Liu et al., 2012). Enforcement of aflatoxin regulatory limits result in loss of markets and reduced income (Van Egmond et al., 2007; Wu, 2014). The most commonly reported aflatoxin producers are *Aspergillus flavus* (produces only B aflatoxins) and *Aspergillus parasiticus* (produces both B and G aflatoxins) (Horn and Dorner, 1998). However, several closely related taxa are known to contaminate crops in Africa including *Aspergillus minisclerotigenes*, *Aspergillus aflatoxiformans*, and an unnamed taxa associated with lethal aflatoxicosis in Kenya (Cotty et al., 2008; Probst et al., 2010; Frisvad et al., 2019; Singh and Cotty, 2019).

*Aspergillus flavus* and other aflatoxin–producers are frequently placed into one of two morphotypes based on sclerotial morphology. The L strain morphotype produces few large sclerotia (average diameter > 400 μm) and the S strain morphotype produces numerous small sclerotia (average diameter < 400 μm) (Cotty, 1989). Fungi with S morphology frequently produce large quantities of aflatoxins and DNA based phylogenetic evidence suggests these aflatoxin-producers belong to several species: (a) *A. flavus* S strain; (b) Lethal Aflatoxicosis Fungus (LAF) S_B_ that led to many deaths in Kenya (Probst et al., 2007); (c), *A. aflatoxiformans* (Cotty and Cardwell, 1999; Frisvad et al., 2019; Singh and Cotty, 2019); and (d) *A. minisclerotigenes* (Pildain et al., 2008).

Aflatoxin-producers infect and produce aflatoxins on both domestic and wild plant species (Boyd and Cotty, 2001). Differences exist among domesticated plants in susceptibility to both aflatoxin-producers and aflatoxin contamination (Mehl and Cotty, 2013a; Kachapulula et al., 2017a) and such variation is also expected among plants that have not been domesticated. Knowledge of plant species vulnerability to aflatoxin contamination may both inform aflatoxin mitigation efforts and facilitate the shaping of diets to limit aflatoxin exposure. In addition, aflatoxin-producer genotypes differ in aflatoxin-producing potential and the relative importance of specific etiologic agents may depend on region (Probst et al., 2007; Cotty et al., 2008). Aflatoxin levels and frequencies of aflatoxin-producers in wild fruits of Zambia have not been characterized.

In order to ascertain levels of aflatoxins and potential for contamination in wild fruits in Zambia, this study: (1) Quantified aflatoxins in the wild fruits *P. curatellifolia*, *Ziziphus* spp., *S. rautanenii*, *Tamarindus indica*, *Vangueriopsis lanciflora*, *Thespesia garckeana and A. digitata*; (2) Characterized communities of *Aspergillus* section *Flavi* in the fruits; and (3) Assessed suitability of the fruits to support growth and aflatoxin production by aflatoxigenic *Aspergillus* section *Flavi*.

## Materials and Methods

### Sampling

**FIGURE 1:**
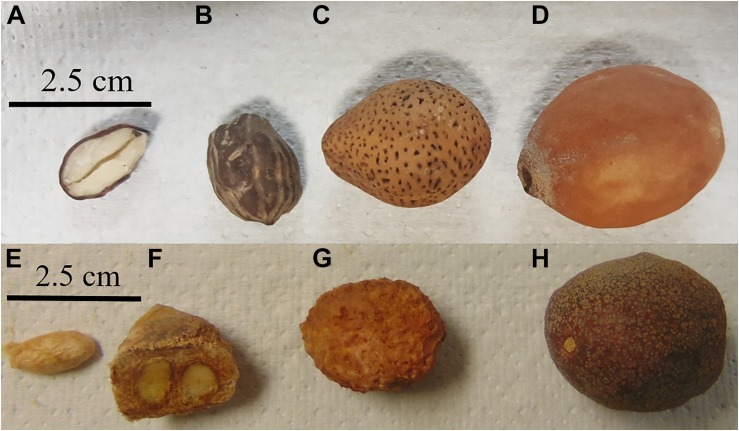
*Schinziophyton rautanenii* **(A–D)** and *Parinari curatellifolia* **(E–H)** from markets in Zambia. **(A)** Cross-section of seed, **(B)** full seed, **(C)** fruit without pulp, **(D)** fruit with pulp, **(E)** seed, **(F)** cross-section of fruit showing characteristic two seeds, **(G)** fruit without pulp, and **(H)** fruit with pulp.

**FIGURE 2:**
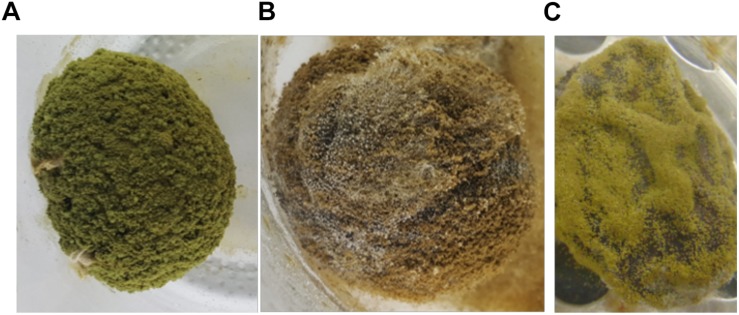
Uncultivated fruits in markets. **(A)** *Thespesia garckeana* in 500 ml (silver) and 200 ml (yellow) containers, **(B)** *Adansonia digitata*, **(C)** *Vangueriopsis lanciflora*, and **(D)** Piles of *Tamarindus indica*.

Samples of dried fruits (107 total) consisting of *S. rautanenii* (*n* = 22), *P. curatellifolia* (*n* = 17) *V. lanciflora* (*n* = 7), *Ziziphus* spp. (*n* = 12), *T. indica* (*n* = 23), *A. digitata* (*n* = 9) and *T. garckeana* (*n* = 17, Table 2 and Figures 1, 2) were collected from markets in 9 districts: Lusaka, Kaoma, Mongu, Senanga, Kapiri Mposhi, Mazabuka, Choma, Livingstone, and Sesheke. For each plant species, up to five fruit samples (350 to 500 g each, with multiple individual fruits in each fruit sample) were obtained from each market where the fruits were present with at least three markets sampled in each district. The fruits were dried at the University of Zambia in a forced air oven (40°C) to 5–8% water content, to prevent fungal growth, and sealed in plastic bags to prevent rehydration. The remainder of the analyses were performed at the USDA, ARS, Laboratory in the School of Plant Sciences, The University of Arizona.

**TABLE 2:** Aflatoxin in non-cultivated fruits purchased from local markets in Zambia.

**Species**	**Samples (#)**	**Aflatoxin (μg/kg)**		**Samples in categories (%)**			
		**Mean**	**Range^a^**	**<4 μg/kg**	**4–9.9 μg/kg**	**10–19.9 μg/kg**	**>20 μg/kg**
*Adansonia digitata*	9	4^BC^	ND-7.5	66.7	33.3	0	0
*Parinari curatellifolia*	17	6^BC^	ND-8.6	29.4	70.6	0	0
*Schinziophyton rautanenii*	22	57^A^	3.4–128.6	4.5	13.6	9.1	72.8
*Tamarindus indica*	23	3^C^	ND-9.0	78.3	21.7	0	0
*Thespesia garckeana*	17	11^B^	3.9–23.2	5.9	41.2	47.0	5.9
*Vangueriopsis lanciflora*	7	12^B^	6.6–18.9	0	28.6	71.4	0
*Ziziphus* spp.	10	6^BC^	ND-24.4	70.0	20.0	10.0	0

*Samples were composed of multiple fruits and weighted 300 to 500 g. Means followed by the same letter (A/B/C) are not significantly different (*P* < 0.05) by Tukey–Kramer’s HSD test. Limit of detection = 2 μg/kg, Range of detection is 2–150 μg/kg. ^*a*^ND = non-detected.*

### Aflatoxin Quantification

Total aflatoxins were quantified with a GIPSA approved lateral flow immunochromatographic assay (Reveal Q + for Aflatoxin, Neogen Corporation, Lansing, MI, United States) following modifications to the manufacturer’s instructions recommended by GIPSA. Briefly, each entire dry fruit sample (350 to 500 g) was ground with either a knife mill (Retsch GM200, Retsch GmbH, Haan, Germany) or cutting mill (Retsch SM100, Retsch GmbH, Haan, Germany) to pass at least 75% of the material through a 20 mesh sieve, mixed thoroughly, and a 50 g sub-sample was blended with 250 ml of 65% ethanol. Aflatoxin content was determined according to the manufacturer’s instructions on a dry weight basis. The aflatoxin quantification technique used was not designed for wild fruits, and as such, results were corrected by spike and recovery experiments done for each fruit. Briefly, ground fruit (5 g) with no detectable aflatoxin was spiked to 100 μg/kg of total aflatoxin using an aflatoxin standard (in methanol, Supelco, Bellefonte, PA, United States). Total aflatoxin was extracted and quantified as described above. Spike and recovery was performed in five replicates. Recovery rates were estimated using the following equation: % Recovery = Total aflatoxin concentration measured in spiked sample/Spiked concentration × 100. Precision of the analytical method was expressed as relative standard deviation (RSD) of replicated results. Recovery rates ranged from 40–60%. Obtained aflatoxin concentrations were corrected to recovery. The limit of detection for Reveal Q + for Aflatoxin is 2 μg/kg and the range of detection is 2–150 μg/kg.

### Isolation and Identification of Fungi From Fruits

Dried fruit samples were ground in either a knife mill (*T. indica*, *Ziziphus* spp., *A. digitata*, *V. lanciflora*, Grindomix GM200, Retsch GmbH, Haan, Germany) or cutting mill (*S. rautanenii*, *T. Garckeana*, and *P. curatellifolia*; Retsch SM100, Retsch GmbH, Haan, Germany) to pass a #12 sieve, and homogenized. Fungi were isolated from ground fruit material using dilution plate technique on modified rose Bengal agar (Cotty, 1994). Briefly, ground fruit material (0.1 to 10 g) was shaken in 50 ml sterile distilled water (20 min, 100 rpm) on a reciprocal shaker (KS-501, IKA Works Inc., Wilmington, NC, United States). Dilution plating was performed in triplicate. Plates were incubated (3 days, 31°C, dark) and up to eight colonies of *Aspergillus* section *Flavi* per isolation were transferred to 5–2 agar (5% V8-juice; 2% agar, pH 5.2). Fungi were stored in sterile water (2 ml) as plugs of sporulating culture after incubation for 7 days at 31°C (Cotty, 1988). Isolations were performed a minimum of twice to obtain a total of at least 15 isolates from each sample. *Aspergillus* species and strains were identified using both macroscopic and microscopic characteristics (Klich and Pitt, 1988; Cotty, 1989, 1994; Probst et al., 2007).

### Wild Fruits as Substrate for Growth and Aflatoxin Production

To evaluate ability of wild fruit to support growth and aflatoxin contamination, colony forming units (CFU) and aflatoxin concentrations were measured on fruit previously inoculated with aflatoxigenic *Aspergillus* section *Flavi*. Briefly, five isolates representing the *A. flavus* L strain morphotype (AF13 = ATCC 96044 = SRRC 1273) and the *A. flavus* S strain morphotype (AF70 = ATCC MYA384), *A. parasiticus* (NRRL 2999), *A. minisclerotigenes* (A-11611) and *A. aflatoxiformans* (A-11612) were inoculated onto sterile whole fruits (10 g in 250 ml Erlenmeyer flask) previously autoclaved for 20 min, cooled to room temperature and moisture adjusted to 30%. The five isolates were chosen because they represent the main aflatoxin-producing species reported to cause aflatoxin contamination in Africa and have frequently been isolated from crops and soils in Africa where the wild fruits under study grow. Spore suspensions containing one million freshly harvested spores from 7-day-old cultures were used as inoculum. After incubation (7 days, 100% RH, 31°C), 100 ml of 0.1% Tween 80 sterile distilled de-ionized water was added and flasks were shaken (650 rpm, mini orbital shaker, Troemner LLC, Thorofare, NJ, United States) for 10 min to wash the conidia from the intact fruits. The resulting conidial suspension was subjected to a 10-fold dilution series and plated onto rose Bengal agar in five replicates. The amount of conidia produced per gram of substrate were expressed on a dry weight basis. To quantify aflatoxin production, cultures were blended in 50 ml of 70% methanol (20 s, maximum speed, Waring 7012S, Waring, Torrington, Connecticut). The slurry was allowed to settle (20 min) and 4 μl of the supernatant was spotted directly onto thin-layer chromatography (TLC) plates (Silica gel 60, EMD, Darmstadt, Germany) adjacent to aflatoxin standards (Aflatoxin Mix Kit-M, Supelco, St. Louis, MO, United States) containing known quantities of aflatoxins B_1_, B_2_, G_1_, and G_2_. Plates were developed in 96:3:1 ethyl ether-methanol-water, air-dried, and aflatoxins were visualized under 365-nm UV light. Aflatoxins were quantified directly on TLC plates using a scanning densitometer (TLC Scanner 3, Camag Scientific Inc., Wilmington, NC, United States) running winCATS 1.4.2 (Camag Scientific Inc., Wilmington, NC, United States). The limit of detection and range of detection for the aflatoxin quantification technique used was 5 μg/kg and 5–500,000 μg/kg, respectively. Recovery rate for this technique was not determined.

### Data Analysis

Aflatoxin concentrations in market samples and aflatoxins produced on fruits in laboratory assays were quantified in micrograms per kilogram (μg kg^–1^). Total quantity of section *Flavi* fungi from each assay was calculated as CFU per gram (CFU g^–1^). Compositions of section *Flavi* communities were described as the percent of the isolates obtained from each fruit sample composed of the *A. flavus* L strain morphotype (Cotty, 1989), un-delineated S strain morphology fungi (Probst et al., 2007), *A. parasiticus* and *Aspergillus tamarii*. Comparisons of both aflatoxin concentrations and fungal populations were performed by Analysis of Variance using general linear models (GLM) and Tukey’s HSD mean comparison test as implemented in JMP 11.1.1 (SAS Institute, Cary, NC, United States). Data were tested for normality and, if required, log transformed (aflatoxin and CFU data) to normalize distributions before analyses. All laboratory tests were replicated five times with a complete randomized block design. Percent data were arcsine-transformed to normalize the distributions prior to analyses. However, actual means are presented for clarity. All tests were performed at α = 0.05.

## Results

### Aflatoxin in Fruit

Significant differences were detected among fruits in aflatoxin concentrations (ANOVA, *F*_6__,__98_ = 25.3786, *P* < 0.001), with the highest average aflatoxin (57 μg/kg) in *S. rautanenii* and the lowest in *Thespesia indica* (3 μg/kg, Table 2). More than 80% of *S. rautanenii* samples had aflatoxin concentrations above the regulatory limit for food in Zambia (10 μg/kg), with fruits containing as much as 128 μg/kg (Table 2). *V. lanciflora* and *T. garckeana* also had average aflatoxins above the regulatory limit for Zambia (12 μg/kg and 11 μg/kg, respectively), with 53 and 71% of fruits having aflatoxins concentrations higher than 10 μg/kg, respectively (Table 2). The average aflatoxin concentrations in *P. curatellifolia* (6 μg/kg), *Ziziphus* spp. (6 μg/kg) and *A. digitata* (4 μg/kg) were all below maximum allowable levels in food in Zambia, although 10% of the *Ziziphus* spp. were above the regulatory limit (Table 2).

### Fungi From Ground Fruit

*Aspergillus* section *Flavi* was recovered from all wild fruits and consisted of *A. parasiticus*, *A. flavus* L strain, S morphotype fungi and *A. tamarii* (Table 3). In total 422 fungal isolates were characterized to species and/or morphotype. The overall quantities (CFU/g) of *Aspergillus* section *Flavi* in the fruits varied (ANOVA, *F*_6__,__74_ = 4.7008, *P* < 0.001, Table 3) with the highest in *P. curatellifolia* (56 CFU/g) and the lowest in *T. indica* (3 CFU/g). Overall frequencies of *Aspergillus* section *Flavi* on fruits differed among the fungi (ANOVA, *F*_3__,__24_ = 35.0131, *P* < 0.001), with *A. flavus* L morphotype most frequent (77.5%, *P* < 0.05). When fruit species were considered individually, the *A. flavus* L strain occurred in the greatest concentrations (*P* < 0.05) on each, except for *V. lanciflora*, where frequencies of *A. flavus* and *A. parasiticus* recovered were not significantly different (51.2 and 44.8%, respectively). Frequencies were similar among fruit species for S morphotype fungi (ANOVA, *F*_6__,__100_ = 1.0444, *P* = 0.4169) and *A. tamarii* (ANOVA, *F*_6__,__100_ = 0.8472, *P* = 0.5440). Higher frequencies of *A. parasiticus* were also found on *T. garckeana* (38.2%) than all other fruits (*P* < 0.05, Table 3), but similar to that recovered from *V. lanciflora*.

**TABLE 3:** Distribution of fungi of *Aspergillus* section *Flavi* on non-cultivated fruits purchased from local markets in Zambia.

**Wild fruit species**	**Samples (#)**	**%L^a,b^**	**%S**	**%P**	**%T**	**CFU/g^c^**
*Adansonia digitata*	9	81.9^AB^	9.8^A^	8.3^BC^	0^A^	8
*Parinari curatellifolia*	17	87.5^AB^	12.5^A^	0^C^	0^A^	56
*Schinziophyton rautanenii*	24	97.1^A^	2.9^A^	0^C^	0^A^	13
*Tamarindus indica*	25	88.4^AB^	3.6^A^	8^BC^	0^A^	3
*Thespesia garckeana*	20	45.3^C^	13.9^A^	38.2^A^	2.7^A^	10
*Vangueriopsis lanciflora*	7	51.2^BC^	0^A^	44.8^A^	0^A^	5
*Ziziphus* spp.	12	91.3^AB^	8.7^A^	0^C^	0^A^	38
Average		77.5^X^	7.3^Y^	14.2^Y^	0.4^Y^	19

*^*a*^L, S, P, and T represent *A. flavus* L morphotype, S morphotype fungi, *A. parasiticus* and *A. tamarii*, respectively. ^*b*^Percent data were arcsine transformed and CFU/g data were log transformed prior to analyses but actual means are presented. Means followed by a common letter (A/B/C) do not differ significantly among fruits (columns) by Tukey’s HSD test (α = 0.05). Average frequencies of fungi across all fruit species followed by a common letter (X/Y) do not differ significantly by Tukey’s HSD test (α = 0.05). ^*c*^Average Colony Forming Units of *Aspergillus* section *Flavi* per gram. *T. indica* is significantly lower (by Tukey’s HSD test, α = 0.05) than *T. garckeana*, *P. curatellifolia*, and *Ziziphus* spp. There are no other significant differences.*

### Wild Fruits as Substrate for Growth by Aflatoxin-Producers

Wild fruits differed in ability to support aflatoxin-producer growth as measured by spore production on fruit surfaces (ANOVA, *F*_5__,__23_ = 176.2224, *P* < 0.001), with the highest average spore production on *V. lanciflora* (1.1 × 10^9^ CFU/g) and no propagules detected on *T. indica* (Table 4). Average spore production across all fruits did not differ among fungi (*F*_4__,__24_ = 0.0045, *P* > 0.05). There were significant differences (ANOVA, *F*_5__,__24_ = 1740.5680, *P* < 0.001) in *A. parasiticus* NRRL 2999 growth among fruit, with the most propagules produced on *V. lanciflora* (6.1 × 10^8^ CFU/g) (Table 4). Similarly, the *A. flavus* S morphotype (AF70; ATCC MYA384) and (A-11612) produced the most propagules on *V. lanciflora* (1.7 × 10^9^ and 1.9 × 10^9^ CFU/g, respectively) (Table 4). On the other hand, *A. minisclerotigenes* (A-11611) sporulated most on *T. garckeana* (6.5 × 10^8^ CFU/g). None of the fungi produced propagules on *T. indica* (Table 4).

**TABLE 4:** Propagules produced by five *Aspergillus* section *Flavi* fungi on inoculated, non-cultivated fruits, gathered in Zambia and purchased in local markets.

**Wild fruit Species**	**Growth of aflatoxin-producers on fruits (CFU/g)**					
	
	***A. parasiticus*^a^**	***A. flavus*-S**	***A. flavus*-L**	***A. minisclerotigenes***	***A. aflatoxiformans***	**Average**
*Parinari curatellifolia*	1.0 × 10^8B(X)^	8.1 × 10^7B(X)^	4.6 × 10^7B(Y)^	1.5 × 10^8B(X)^	9.1 × 10^7B(X)^	9.4 × 10^7C^
*Schinziophyton rautanenii*	1.2 × 10^8B(X)^	2.3 × 10^6C(Z)^	2.7 × 10^8A(X)^	5.7 × 10^7C(Y)^	3.1 × 10^7C(Y)^	9.5 × 10^7C^
*Tamarindus indica*	ND^b^	ND	ND	ND	ND	ND
*Thespesia garckeana*	1.6 × 10^7C(Z)^	8.7 × 10^7B(Y)^	2.2 × 10^8A(X)^	6.5 × 10^8A(W)^	1.3 × 10^8B(Y)^	2.2 × 10^8B^
*Vangueriopsis lanciflora*	6.1 × 10^8A(Y)^	1.7 × 10^9A(X)^	N/A	1.5 × 10^8B(Z)^	1.9 × 10^9A(X)^	1.1 × 10^9A^
*Ziziphus* spp.	2.0 × 10^6D(Y)^	7.3 × 10^7B(X)^	7.3 × 10^7B(X)^	4.4 × 10^6D(Y)^	7.3 × 10^6D(Y)^	3.2 × 10^7C^
Average	1.7 × 10^8X^	4.0 × 10^8X^	1.5 × 10^8X^	2.0 × 10^8X^	4.4 × 10^8X^	

*^*a*^*Aspergillus parasiticus* = Isolate NRRL 2999, *A. flavus-S* = Isolate AF70/ATCC MYA384, *A. flavus-*L = Isolate AF13/ATCC 96044/SRRC 1273, *A. minisclerotigenes* = Isolate A-11611, *Aspergillus aflatoxiformans* = Isolate A-11612. Means followed by the same letter (A/B/C) among fruits (columns) or (W/X/Y/Z) among fungal species (rows) were not significantly different (*P* < 0.05) by Tukey–Kramer’s HSD test. ^*b*^ND = not detected.*

### Wild Fruits as Substrate for Aflatoxin Production

With the exception of *T. indica*, all fruit species supported production of aflatoxin concentrations greater than 10 mg/kg during the 7-day growth period (Table 5). On average, there were significant differences among fruits in concentrations of aflatoxins produced (ANOVA, *F*_5__,__24_ = 61.9592, *P* < 0.001), with the highest concentrations produced on *T. garckeana* (average = 73,500 μg/kg, Table 5). On average across fruit species, concentrations of aflatoxins produced by the six *Aspergillus* section *Flavi* did not differ (ANOVA, *F*_4__,__25_ = 0.0215, *P* = 0.9990; Table 5). Among the fungi examined, *A. parasiticus* produced the highest concentrations of aflatoxins on *S. rautanenii* (182,000 μg/kg) and *T. garckeana* (131,000 μg/kg) with *A. aflatoxiformans* producing statistically similar quantities (mean = 212,000) on *T. garckeana*; Table 5). *A. parasiticus* produced less aflatoxins on *Ziziphus* spp. (16,100 μg/kg), *P. curatellifolia* (5,081 μg/kg), *V. lanciflora* (2,729 μg/kg) or *T. indica* (*P* < 0.05). *A. flavus* L (AF13), and S (AF70) morphotypes produced similar concentrations on fruits (Table 5). *A. minisclerotigenes* was the most aflatoxigenic species on *Vangueriopsis* (76,604 μg/kg; Table 5).

**TABLE 5:** Ability of non-cultivated fruits, gathered in Zambia and purchased in local markets, to support aflatoxin production by five aflatoxin producers.

**Non-cultivated fruit genus**	**Aflatoxin production (μg/kg)**					
	
	**^a^*A. parasiticus***	***A. flavus*-S**	***A. flavus*-L**	***A. minisclerotigenes***	***A. aflatoxiformans***	***Average***
*Parinari curatellifolia*	5,081^BC(X)^	1,517^A(X)^	14,655^A(X)^	7,950^AB(X)^	5,573^B(X)^	6,955^A^
*Schinziophyton rautanenii*	182,214^A(X)^	17,256^A(Y)^	6,356^A(Y)^	31,407^AB(XY)^	19,109^B(Y)^	51,268^A^
*Tamarindus indica*	0^D^	0^B^	0^B^	0^C^	0^C^	0^B^
*Thespesia garckeana*	131,065^A(X)^	9,843^A(Y)^	9,395^A(Y)^	4,730^B(Y)^	212,596^A(X)^	73,526^A^
*Vangueriopsis lanciflora*	2,729^C(X)^	15,687^A(XY)^	33,336^A(XY)^	76,604^A(X)^	5,594^B(XY)^	26,790^A^
*Ziziphus*	16,060^B(X)^	39,222^A(X)^	11,946^A(X)^	4,338^B(X)^	20,113^B(X)^	18,336^A^
Average	56,192^X^	13,921^X^	12,615^X^	20,839^X^	43,831^X^	

*^*a*^*Aspergillus parasiticus* = Isolate NRRL 2999, *A. flavus-S* = Isolate AF70/ATCC MYA384, *A. flavus-*L = Isolate AF13/ATCC 96044/SRRC 1273, *A. minisclerotigenes* = Isolate A-11611, *A. aflatoxiformans* = Isolate A-11612. Values are means of the results of analyses on five independent solid fermentations (replicates) incubated for 7 days at 31°C. Means followed by the same letter (A/B/C) among fruits (columns) or (X/Y) among fungal species or morphotypes were not significantly different (*P* < 0.05) by Tukey–Kramer’s HSD test.*

## Discussion

In the current study, aflatoxins were detected in all the examined fruit species (Table 2) with distributions similar to that previously reported for non-cultivated fruits gathered in North America (Boyd and Cotty, 2001). The current report is the first documentation of aflatoxins in wild tropical fruits including *S. rautanenii*, *V. lanciflora*, *T. garckeana*, *P. curatellifolia*, *Ziziphus* spp., and *A. digitata*. Although aflatoxin recovery rates are low, our results provide an initial indication that these fruits are a source of aflatoxin exposure and support development of substrate specific methods that might allow more precise quantification of the extent of human exposure resulting from actual consumption patterns. As is the case with crop plants, susceptibility to aflatoxin contamination in wild fruits varies among species (Boyd and Cotty, 2001; Table 2). Samples of *S. rautanenii* collected during the current study contained aflatoxin concentrations exceeding 125 μg/kg, whereas, all *T. indica* contained <10 μg/kg total aflatoxins. Differences among fruits in concentrations of aflatoxins may be attributable to chemical composition (Mehl and Cotty, 2013b), variation in structures of communities of *Aspergillus* section *Flavi* colonizing the fruits (Mehl et al., 2012), and/or fruit specific processing and storage practices. Although it is not known whether the aflatoxins detected in *S. rautanenii* were primarily in the seed or pulp, both components of the fruit are important in human diet and would contribute to aflatoxin exposure in Zambia.

*Aspergillus* section *Flavi* community structure influences aflatoxin-producing potential of fungi infecting crops and, as a result, the extent of contamination (Probst et al., 2007; Mehl et al., 2012). In wild fruits of Zambia, the *A. flavus* L morphotype was the most frequent (77.5%) member of *Aspergillus* section *Flavi* (Table 3). High frequencies of *A. flavus* L morphotype on fruit are not surprising because even though soils in forests and cultivated areas of Zambia are dominated by *A. parasiticus* (Kachapulula et al., 2017b), the *A. flavus* L morphotype is a much more competitive plant colonizer. It was unexpected that high amounts of aflatoxins in *S. rautanenii* were associated with high frequencies of the *A. flavus* L morphotype because, most *A. flavus* L morphotype from Zambia are either atoxigenic or have low aflatoxin-producing potential (Kachapulula et al., 2017a). Similarly, higher levels of aflatoxins were expected in *P. curatellifolia*, due to incidences of over 10% of the high aflatoxin-producing S morphology fungi (Table 3). It is possible that *S. rautanenii* selects for aflatoxigenic *A. flavus* L morphotype (Sweany et al., 2011). However, it is more likely that environmental conditions between fruit development and collection predisposed *S. rautanenii* to high aflatoxin concentrations and *P. curatellifolia* to low concentrations. On the other hand, *V. lanciflora* and *T. garckeana* had appreciable levels of S morphotype fungi and/or *A. parasiticus* (Table 3) and as expected (Cotty et al., 2008), high concentrations of aflatoxins (Table 2).

The suitability of fruits gathered from the wild as substrates for growth of aflatoxigenic fungi was evaluated by quantifying propagule production by reference aflatoxigenic fungi. All tested fruits supported growth except *T. indica* (Table 4). Both cultivated (Mehl and Cotty, 2013a) and non-cultivated plants (Boyd and Cotty, 2001) are susceptible to infection by aflatoxin-producing fungi. Invasion of host tissues requires plant-degrading enzymes, ability to evade or suppress host defenses, and capacity to overcome plant antimicrobial compounds (Agrios, 2005). In the current study, *V. lanciflora* supported the greatest growth by all aflatoxigenic fungi. On the other hand, even though low amounts of *Aspergillus* section *Flavi* were found in *T. indica* market samples (Table 3), growth by the reference aflatoxin-producers on *T. indica* was not detected (Table 4). Phenolic antioxidants from *T. indica* have antifungal properties (Luengthanaphol et al., 2004; Sudjaroen et al., 2005). These antioxidants may be responsible for low fungal growth on *T. indica*. *Tamarindus indica* is also known to contain a number of organic acids, including tartaric acid, which causes the fruit’s characteristic sour taste (Rao and Mathew, 2012). Tartaric acid is known to have antifungal activity against several species of fungi, including *A. flavus* (Hassan et al., 2015). Tartaric acid and other organic acids may have contributed to the low fungal growth observed in the current study on *T. indica*. Results of the current study suggest that *T. indica* is a low risk aflatoxin food. However, all other examined wild fruits supported growth by aflatoxin-producers and therefore potential measures for aflatoxin mitigation might include strategies for preventing fungal growth.

Aflatoxin-producers may grow on substrates without producing aflatoxins. Aflatoxin production is dependent on environmental conditions (Cotty and Jaime-Garcia, 2007; Kachapulula et al., 2017a) and substrate nutritional content (Mehl and Cotty, 2013b). Fruit suitability for growth of aflatoxin-producers contributes to fruit vulnerability. However, the quantity of *Aspergillus* section *Flavi* on market samples was not significantly correlated with the quantity of aflatoxins in those fruits (unpublished observations). This suggests factors other than just support of growth of *Aspergillus* section *Flavi*, such as environmental conditions, toxigenicity of infecting fungi and/or handling (Cotty and Jaime-Garcia, 2007; Kachapulula et al., 2017a), dictated the extent of contamination observed in the wild fruits during the study period in Zambia.

**FIGURE 3:**
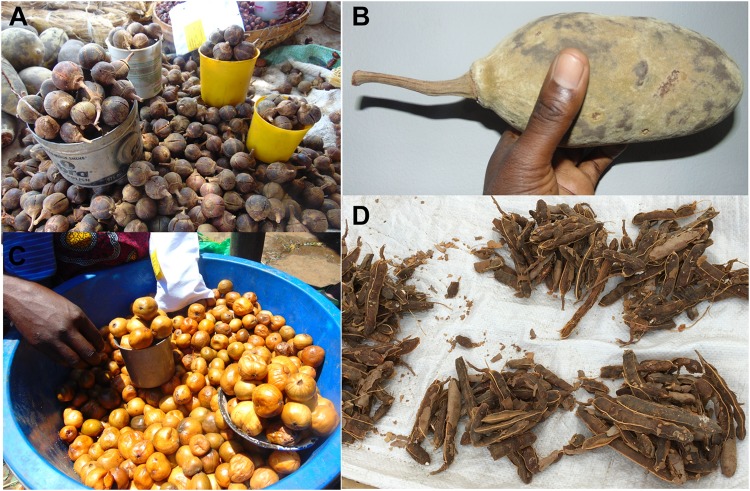
Growth of *Aspergillus* section *Flavi* on inoculated fruits. **(A)** *Aspergillus flavus* L. morphotype on *Parinari curatellifolia*, **(B)** *Aspergillus parasiticus* on *Thespesia garckeana*, and **(C)** *Aspergillus minisclerotigenes* on *Vangueriopsis lanciflora*. All other fungi had similar growth on all fruits except on *Tamarindus indica*, which did not support fungal growth during these tests.

Inoculation of fruits with known aflatoxin-producers under conditions conducive for aflatoxin production provides insight into innate capacity to become contaminated. The fruits varied widely in ability to support aflatoxin biosynthesis and the fruit most vulnerable to contamination was dependent on the aflatoxin-producing species. One fruit (*T. indica*) supported no aflatoxin production in laboratory inoculation experiments. However, very low concentrations (<10 μg/kg) were detected in market samples. So there is some risk of aflatoxin exposure associated with consumption of *T. amarindus*. Aflatoxins in market *Tamarindus* may reflect conditions not evaluated in the inoculation tests including aflatoxin formation during fruit development, in association with insect damage, or as a result of long periods of poor storage (Boyd and Cotty, 2001; Cotty et al., 2008). The other species evaluated during laboratory inoculation experiments became contaminated with very high concentrations (Table 5). Even fruits for which market samples were safe for consumption (*P. curatellifolia* and *Ziziphus* spp., Table 2) developed high (>6,000 and >18,000 μg/kg, respectively) concentrations of aflatoxins after inoculation with aflatoxin-producers (Table 5). The second largest concentrations of aflatoxin observed (over 180,000 μg/kg total aflatoxins) resulted from inoculation of the most frequently contaminated fruit from the market (*S. rautanenii*) with *A. parasiticus*, the most common aflatoxin producer in Zambia (Kachapulula et al., 2017b). The results suggest that assessment of fruit vulnerability to contamination should include assessment of both frequencies of specific aflatoxin-producers in the habitat and the suitability of the fruit for contamination by the resident fungi. An additional element important in evaluating fruit vulnerability to contamination not investigated in the current study, is anatomical change during fruit development. In pistachio, early splitting of the hull results in higher quantities of aflatoxins and aflatoxin-producing fungi (Doster and Michailides, 1994; Hadavi, 2005). In almonds, amounts of fungi associated with the crop are highest during hull splitting (Ortega-Beltran et al., 2018). It is possible that for some fruit species additional entry points were created during postharvest handling and the laboratory inoculation process, resulting in increases in vulnerability to the contamination process. This further emphasizes the need for careful fruit handling after harvest. For other fruits examined in the current study, such as *T. indica*, the laboratory handling did not result in aflatoxin increases suggesting that regardless of handling, the fruit retained low susceptibility to aflatoxin contamination. The current study was the first to quantify both the infecting fungi and the ability of fruit from Zambia’s diverse forest to market system to support aflatoxin biosynthesis. Although consumers will typically not pick and eat fruits with profuse fungal growth as in Figure 3, the current study indicates many wild fruits support growth and aflatoxin production, and as such, proper handling of the foods is needed.

Aflatoxin-producing potentials of infecting fungal communities may have modulated contamination of wild fruits of *P. curatellifolia* and *Ziziphus* spp. in the current study. *P. curatellifolia* and *Ziziphus* spp. supported high aflatoxin concentrations when inoculated, but fruits collected from markets were also most frequently infected by the *A. flavus*-L morphotype (Table 3), which in Zambia, is primarily either atoxigenic or of low aflatoxigenicity (Kachapulula et al., 2017a). The low aflatoxin content observed in market samples of these two fruit species thus does not result from innate resistance. The low aflatoxin concentrations may result from a combination of safe handling and storage (Kachapulula et al., 2017a), limited entry points for aflatoxin-producing fungi (Doster and Michailides, 1994; Hadavi, 2005) and a fungal community with low average aflatoxigenicity (Probst et al., 2007, 2010; Cotty et al., 2008). The use of atoxigenic biocontrol agents to modulate *Aspergillus* fungal communities associated with fruits, as has been done in pistachios and almonds (Cotty et al., 2007; Bandyopadhyay et al., 2016), might reduce incidences of aflatoxin-producers and resulting aflatoxin contamination, even where natural anatomical openings in fruits are unavoidable.

In the current study, with the exception of *T. indica*, all wild fruits either had aflatoxin levels above regulatory limits for Zambia, or were suitable substrates for growth and aflatoxin production by aflatoxigenic fungi. Reducing concentrations of aflatoxins in consumed wild fruits may be an important aspect of minimizing overall aflatoxin exposure for Zambian populations. Aflatoxin concentrations in some fruits appear to be a result of environmental conditions and infecting fungi, while for others, innate immunity appears to be the major contributor to contamination patterns. Aflatoxin concentrations in gathered fruits can be limited by not collecting insect damaged fruits, avoiding fruits with conspicuous openings, picking from bearing plants rather than collecting off the ground, and synchronizing gathering periods to precede periodic events such as annual or biannual rains (Doster and Michailides, 1994; Boyd and Cotty, 2001; Hadavi, 2005; Garber et al., 2013). After collection, aflatoxin concentration increases can be limited by proper drying and dry storage. Future research should focus on developing baseline information and practical culturally acceptable techniques for limiting contamination in non-cultivated fruits that are gathered, marketed, and consumed in Zambia. Future research should also look into the possibility of using atoxigenic *A. flavus* for biological control of aflatoxins, especially where attempts are underway to domesticate wild fruit species. Biological control with atoxigenic strains of *A. flavus* is the most effective control method against aflatoxin contamination (Cotty et al., 2007; Bandyopadhyay et al., 2016). Restricting gathering to areas in close proximity to agricultural fields treated with an atoxigenic strain based biocontrol product may also serve to reduce quantities of aflatoxins associated with the gathered fruits.

## Data Availability

The datasets generated for this study are available on request to the corresponding author.

## Author Contributions

PK, RB, and PC contributed to conception and design of the study. PK performed the statistical analyses and wrote the first draft of the manuscript. All authors contributed to the manuscript revision, and read and approved the submitted version.

## Conflict of Interest Statement

The authors declare that the research was conducted in the absence of any commercial or financial relationships that could be construed as a potential conflict of interest.
